# Scanning Electron Microscopic Studies of the Pecten Oculi in the Quail (*Coturnix coturnix japonica*)

**DOI:** 10.1155/2013/650601

**Published:** 2013-10-02

**Authors:** Aris F. Pourlis

**Affiliations:** Laboratory of Anatomy, Histology & Embryology, Veterinary Faculty, University of Thessaly, 224 Trikalon street, 43100 Karditsa, Greece

## Abstract

The main purpose of this study is to extend the microscopic investigations of the pecten oculi in the quail in order to add some information on the unresolved functional anatomy of this unique avian organ. The pecten oculi of the quail was studied by scanning electron microscopy. Eighteen- to-twenty two highly vascularised accordion-like folds were joined apically by a heavily pigmented bridge of tissue, which holds the pecten in a fanlike shape, widest at the base. The structure of the double layered limiting membrane was recorded. The presence of hyalocytes with macrophage-like appearance was illustrated. It is assumed that the pecten oculi of the quail resembles that of the chicken. Illustrated morphological features of this species may add information on the active physiological role of the pecten. But still, the functional significance of this organ is a matter of controversies.

## 1. Introduction 

The pecten oculi is a unique anatomical structure of the avian eye. Three types of pecten are recognized: the conical type (*pecten oculi conicus*) found only in kiwis, the vaned type (*pecten oculi vanellus*) found in the other extant Struthioniformes, and the tinamous and the pleated type (*pecten oculi plicatus*) found in all other birds [1]. The pecten has been frequently studied and extensive literature about pecten is available [2, 3]. Consequently, many investigations were carried out by light and transmission electron microscopy, regarding the structure of the pecten in various avian species (Table 1). In the great majority of the studies, light and transmission electron microscopy have been used. 

The morphology of the pecten in the quail (*Coturnix coturnix japonica*) has been studied by scanning (SEM) and transmission (TEM) electron microscopy [4]. Moreover, branching of vessels in pecten oculi has also been investigated by light microscopy and stereomicroscopy [5]. However some morphological features such as the hyalocytes or the structure of the pectin-vitreal limiting membrane have not been recorded. 

The present work aims to provide some unexplored morphological data of the pecten of the quail with the use of scanning electron microscopy. In addition, a concise review and discussion aim to summarize the up to date knowledge, in order to throw light on the functional structure of this organ.

## 2. Material and Methods

In the present study, six adult healthy quails of both sexes were used. Prior to decapitation, the animals were deeply anesthetized by inhalation of diethyl ether. The ocular bulbs were immediately removed and opened to facilitate access of the fixative used for immersion fixation. Ten of them were fixed in a sodium cacodylate buffered solution of 2% glutaraldehyde and 2% paraformaldehyde. Subsequently, pieces of the pectens were washed four times, for 15 min each time, with sodium cacodylate buffer (pH 7.2), then transferred for 1 h to 1% OsO_4_, and dehydrated in graded acetone series. Tissues were critical point-dried in carbon dioxide (72–75 Barr), mounted onto stubs, and sputter coated with platinum and gold in a Bal-Tec sputter coater. Specimens were observed in a scanning electron microscope (JEOL, JSM 840).

## 3. Results

**Figure 1:**
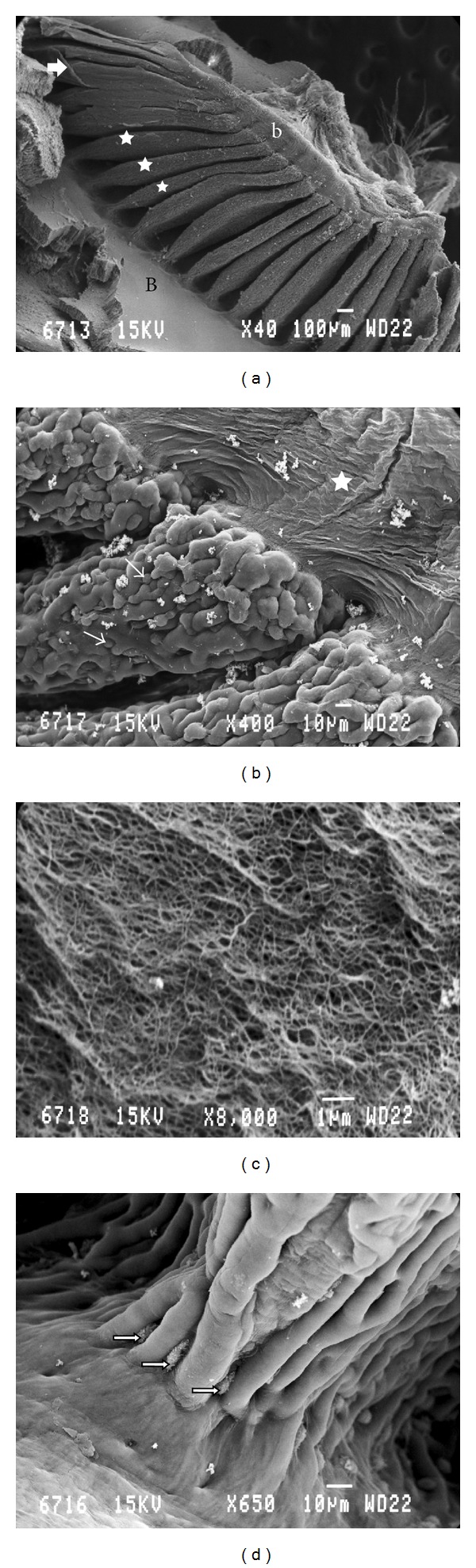
(a) Scanning electron micrograph of the pecten's overview. B: the base of the pecten; b: the bridge of the pecten; arrow: the vitreopectineal membrane; the asterisks denote the pleats. (b) Scanning electron micrograph of three pleats ending at the bridge of the pecten. The asterisk shows the bridge. Thin arrows: pigmented cells. (c) Scanning electron micrograph of the fibrillar tissue of the bridge. (d) Scanning electron micrograph of a pleat arising from the base. Note the presence of hyalocytes (arrows).

**Figure 2:**
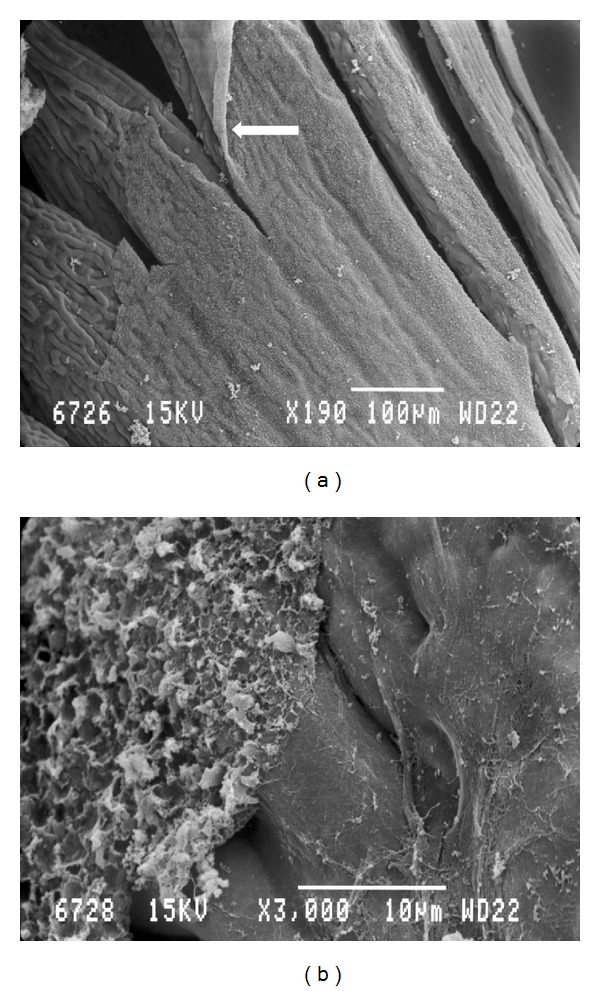
(a) Scanning electron micrograph of the vitreopectineal limiting membrane (arrow). (b) Scanning electron micrograph of the vitreopectineal limiting membrane. Note the outer layer (left side) and the inner layer (right side).

**Figure 3:**
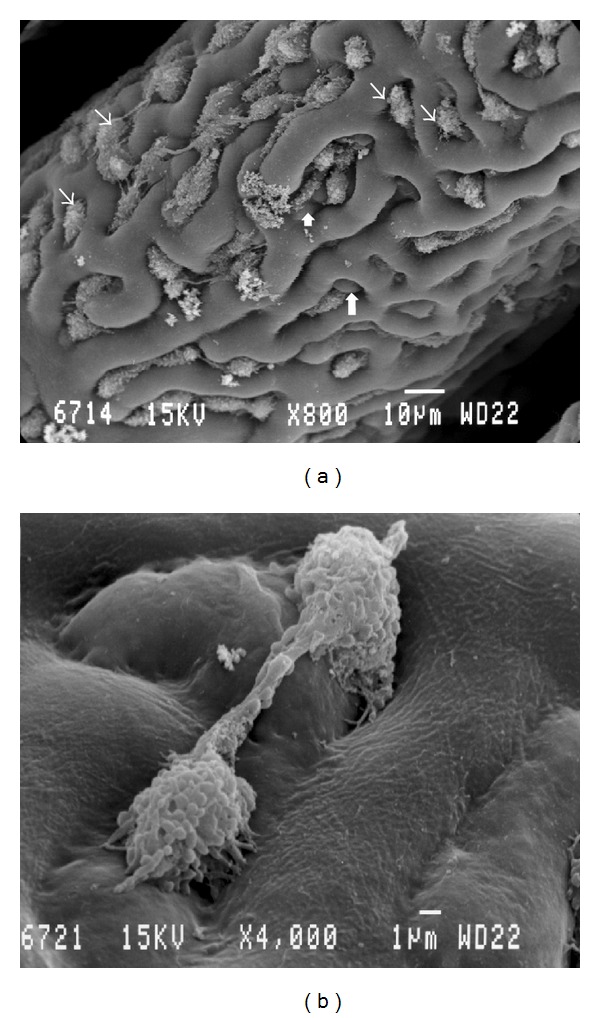
(a) Scanning electron micrograph of the surface of a pleat. Note the presence of the pigmented cells (arrows) and the hyalocytes (thin arrows). (b) Scanning electron micrograph of two hyalocytes.

**Figure 4:**
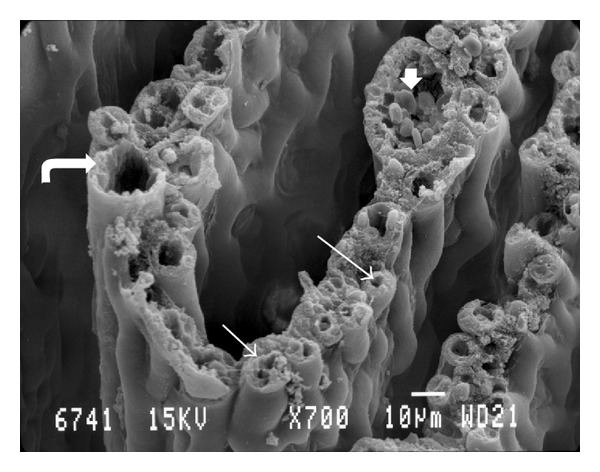
Scanning electron micrograph of various profiles of the pectineal vessels (thin arrows). Curved arrow and arrow head, point to greater (in diameter) vessels.

The pecten of the quail was a fan-like intraocular structure situated over the head of the optic nerve. It was trapezoidal shaped and black in color. Pleated type pecten oculi was comprising 18–22-folds which were joined apically on a fibrous bridge (Figures 1(a), 1(b), and 1(c)). The pleats were attached basally at intervals along the entire length of the linear portion of the optic disc (Figures 1(a) and 1(d)). The pectin was measured 4-5 mm in length at its base, whereas the bridge measured 2.5–3 mm. The mean height and width of pleats were 1 mm and 100 *μ*m, respectively. A superficial membrane surrounding the pecten oculi was separating it from the vitreous body (Figure 2(a)). This structure was composed of two different laminae (Figure 2(b)). The basal lamina surrounded each blood vessel. It was attached on the outer aspect of the blood vessels and contained a large number of delicate reticular microfibrils (Figure 2(b)). The outermost had the appearance of amorphous substance (Figure 2(b)). The surface of the pleats showed a dense vascular network with branching and anastomosing vessels. Within this network extravascular spheroidal bodies were observed and identified as pigmented cells (Figure 3(a)). On the pectineal surface and especially on the pleats, amoeboid phagocyte-like cells, the hyalocytes were found (Figure 3(a)). These cells were not observed at the bridge of the pecten. They were seen isolated, double, or forming clusters both in the external and internal faces of the pleats. The hyalocytes had various shape, size, and number of cytoplasmatic processes. Their length varied from 6 *μ*m to 10 *μ*m whereas their width was 4-5 *μ*m. Their external morphology exhibited undulating membrane with filopodia (Figure 3(b)). The transverse section of the vascular network of the pecten revealed the profile of numerous capillaries with diameter ranging from 5 to 10 *μ*m. Among them, each fold contained two or more larger blood vessels, which were difficult to differentiate as either arterioles or venules (Figure 4). Their diameter frequently exceeded the 20 *μ*m.

## 4. Discussion 

The pecten oculi of the quail (*Coturnix coturnix japonica*), in concert with other avian species, is a pigmented highly vascular, fanlike structure situated over the head of the optic nerve and projecting out into the vitreous. My observations are in agreement with previous studies in the quail [4, 5]. The morphometrical data of this study are in the same range with that of Orhan et al. [5]. The general morphology of the quail's pecten resembles that of chicken [6–8].

The limiting membrane which covers the pecten has been illustrated for the first time in terms of SEM. Besides the numerous morphological investigations of the avian pecten, some of them have illustrated the limiting membrane by means of TEM [7, 9]. Our observations are similar to reports of chicken [7] and pigeons [9]. The authors demonstrated the double layered limiting membrane by TEM. The double layer constitutes a barrier between the pecten and the vitreous body.

The presence of hyalocytes on the pectineal surface of the quail has been demonstrated for the first time by means of SEM, in the present study. Previous investigations in the developing quail revealed the phagocytic activity of the hyalocytes [10]. These specialized cells have also been observed in the chicken [8], the mallard [11], and the budgerigar [12]. In contrary, they have not been observed in many other avian species [2, 9, 13]. Fischlschweiger and O'Rahilly [7] described with means of TEM the presence of cells on the external aspect of the covering membrane. These cells, termed “peripectinate cells,” were thought by the authors to be a special type of astroglia. As these cells display phagocytosis and from their histochemistry, it has been concluded that the hyalocytes are a subtype of macrophage [8]. My observations agree with the positive relationship between the distribution of hyalocytes and blood vessels in the chicken [8]. In fact, the hyalocytes were numerous on the free surface of the pleats excluding the bridge in which the relatively fewer vessels were located in the deep portion. Llombart et al. [10] proposed that hyalocytes might prevent the movement of macromolecules into the retina by their macrophagic activity. 

The presence of pigmented cells is a constant feature of all pectens recorded to date. In all species described, there is a marked increase in melanocytes in the apex or bridge region of the pecten [2]. Overlying and within the vascular network, a close association between blood vessels and pigmented cells is evident. This association may serve as a protective mechanism of the vessels against the effects of ultraviolet light [12, 14].

Fine structural studies to date indicate that while the pleated pecten was widespread and essentially similar in most species, there were some variations in such parameters as shape, size, number of folds, and the thickness of capillary basal laminae [15]. The number of pleats varies among the species which possess a pleated type pecten (Table 1). SEM studies showed the approximate number of pleats was 18–22 in the chicken [6, 23]. In the present study the number ranged from 18 to 22 similar to chicken and to quail [4, 5]. Many attempts have been made to correlate the number of pleats with the function of the pecten. From a survey of the existing literature (Table 1) no correlation could be found regarding the number and the zoological classification of the birds. The number of pleats in the quail is similar to the fowl [6, 23]. Both of them have a zoological closeness since they belong to the same family of Phasianidae. On the other hand, the budgerigar [12] and the Australian galah [21] which belong to the family of Psittacidae have 10–12 and 20–25 pleats, respectively. The size of the pecten and the number of pleats do not appear to relate directly to the size of the eye itself. On the other hand, the size of the pecten and the number of pleats seem to pertain to the behavior of the bird in relation to its general activity and its environmental lighting. Furthermore, these variations seem to correlate with the diurnal activity and/or visual requirements of the species (Table 1). The same deduction has been recorded by Pettigrew [31] who compared the number of pectineal pleats of 8 diurnal and 8 nocturnal birds. However this correlation may not be evidential; for example, the emu has a pecten with 3-4 pleats [20]. 

Despite the numerous publications, the functional significance of the pecten remains controversial. It is more than a century ago when the function of the pecten has challenged the imagination of investigators. The pecten according to numerous authors is mainly concerned with the nourishment of the avascular avian retina [17, 20]. The intriguingly dense meshwork of capillaries points to erectile tissue of the pecten which potentially could serve as sun shade [27]. The double limiting membrane provides the mechanical support of the pecten and a barrier between the pecten capillaries and the hyaloid body. In the same capillaries, blood which is circulated, could serve as carrier of various sensory molecules. The hyalocytes probably serve as sweepers of unwanted material within the hyaloid body [8]. Frost and Mouritsen [32] claimed that the pecten could possibly function as an “ocular sextant.” Maybe the pecten could potentially be engaged in avian navigation. 

## 5. Conclusions

The purpose of the present study was to give some additional information on the morphology of the avian pecten and to explore possible relations between the structure and function of this organ. The appearance of the hyalocytes, as well as the illustration of the bilaminar structure of the covering membrane, was demonstrated for the first time. These morphological characteristics add information on the structure of the pecten oculi. However, there is need for a further approach to throw light in this enigmatic organ.

## Figures and Tables

**Table 1:** Research studies of pecten oculi in various avian species.

Species	Method	Number of pleats	Order	Family	Visual activity	References
Common loon (*Gavia immer*)	LM, TEM	14-15	Gaviiformes	Gaviidae	Diurnal	[15]
Pigeon (*Columba livia*)	LM, TEM	15–17	Columbiformes	Columbidae	Diurnal	[16]
	LM, SEM	15–18				[17]
Mallard (*Anas platyrhynchos*)	LM, TEM	12–14	Anseriformes	Anatidae	Diurnal	[11]
Black kite (*Milvus migrans*)	SEM	12	Falconiformes	Accipitridae	Diurnal	[14]
Red-tailed hawk (*Buteo jamaicensis*)	LM, TEM	17-18	Accipitriformes	Accipitridae	Diurnal	[18]
American crow (*Corvus brachyrhynchos*)	LM, TEM	22–25	Passeriformes	Corvidae	Diurnal	[19]
Nighthawk (*Chordeiles minor*)	LM, TEM	4-5	Caprimulgiformes	Caprimulgidae	Nocturnal	[13]
Emu (*Dromaius novaehollandiae*)	LM, TEM	3-4	Struthioniformes	Dromaiidae	Diurnal	[20]
Australian galah (*Eolophus roseicapillus*)	LM, TEM	20–25	Psittaciformes	Psittacidae	Diurnal	[21]
Great horned owl (*Bubo virginianus*)	LM, TEM	7-8	Strigiformes	Strigidae	Nocturnal	[22]
Chicken (*Gallus domesticus*)	SEM, TEM	18–20	Galliformes	Phasianidae	Diurnal	[23]
	LM, SEM	18–22				[6]
	LM, TEM	16–18				[24]
Great blue heron (*Ardea herodias*)	LM, TEM	14-15	Pelecaniformes	Ardeidae	Diurnal	[25]
Barred owl (*Strix varia*)	TEM	8–10	Strigiformes	Strigidae	Nocturnal	[26]
Common buzzard (*Buteo buteo*)	LM, SEM	17	Falconiformes	Accipitridae	Diurnal	[27]
Budgerigar (*Melopsittacus undulatus*)	LM, SEM, TEM	10–12	Psittaciformes	Psittacidae	Diurnal	[12]
Jungle Crow (*Corvus macrorhynchos*)	LM, TEM	24-25	Passeriformes	Corvidae	Diurnal	[28]
Spotted eagle owl (*Bubo bubo africanus*)	LM, TEM	5-6	Strigiformes	Strigidae	Nocturnal	[24]
White stork (*Ciconia ciconia*)	LM, SEM	15–17	Ciconiiformes	Ciconiidae	Diurnal	[29]
Eurasian sparrowhawk (*Accipiter nisus*)	LM	11-12	Falconiformes	Accipitridae	Diurnal	[30]
Japanese quail (*Coturnix coturnix japonica*)	LM	19	Galliformes	Phasianidae	Diurnal	[5]
	SEM, TEM	18				[4]
